# 2-(4-Bromo­phen­yl)-*N*-(5-methyl­pyridin-2-yl)acetamide

**DOI:** 10.1107/S1600536812032631

**Published:** 2012-07-25

**Authors:** Hoong-Kun Fun, Chin Wei Ooi, Prakash S. Nayak, B. Narayana, B. K. Sarojini

**Affiliations:** a X-ray Crystallography Unit, School of Physics, Universiti Sains Malaysia, 11800 USM, Penang, Malaysia; b Department of Studies in Chemistry, Mangalore University, Mangalagangotri 574 199, India; c Department of Chemistry, P. A. College of Engineering, Nadupadavu, Mangalore 574 153, India

## Abstract

The asymmetric unit of the title compound, C_14_H_13_BrN_2_O, consists of two mol­ecules; the dihedral angles between the pyridine and benzene rings are 87.99 (9) and 84.28 (9)°. An intra­molecular C—H⋯O hydrogen bond generates an *S*(6) ring in each mol­ecule. In the crystal, mol­ecules are linked *via* N—H⋯N and C—H⋯O hydrogen bonds into a three-dimensional network. The crystal structure also features weak π–π stacking inter­actrions between the benzene rings [centroid-to-centroid distance = 3.6829 (12) Å].

## Related literature

For related structures, see: Fun *et al.* (2012*a* ▶,*b* ▶). For hydrogen-bond motifs, see: Bernstein *et al.* (1995 ▶). For the stability of the temperature controller used for the data collection, see: Cosier & Glazer (1986 ▶).
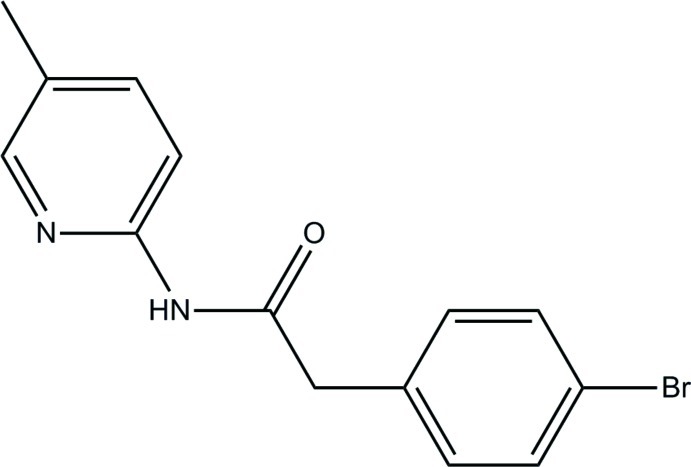


## Experimental

### 

#### Crystal data

C_14_H_13_BrN_2_O
*M* *_r_* = 305.17
Monoclinic, 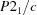

*a* = 14.0086 (16) Å
*b* = 9.4215 (11) Å
*c* = 20.610 (2) Å
β = 109.040 (2)°
*V* = 2571.3 (5) Å^3^
*Z* = 8
Mo *K*α radiation
μ = 3.19 mm^−1^
*T* = 100 K
0.35 × 0.31 × 0.16 mm

#### Data collection

Bruker APEX DUO CCD diffractometer
Absorption correction: multi-scan (*SADABS*; Bruker, 2009 ▶) *T*_min_ = 0.404, *T*_max_ = 0.626
28356 measured reflections
7539 independent reflections
5774 reflections with *I* > 2σ(*I*)
*R*_int_ = 0.049

#### Refinement

*R*[*F* ^2^ > 2σ(*F* ^2^)] = 0.033
*wR*(*F* ^2^) = 0.068
*S* = 1.03
7539 reflections
327 parameters
H-atom parameters constrained
Δρ_max_ = 0.72 e Å^−3^
Δρ_min_ = −0.84 e Å^−3^

### 

Data collection: *APEX2* (Bruker, 2009 ▶); cell refinement: *SAINT* (Bruker, 2009 ▶); data reduction: *SAINT*; program(s) used to solve structure: *SHELXTL* (Sheldrick, 2008 ▶); program(s) used to refine structure: *SHELXTL*; molecular graphics: *SHELXTL*; software used to prepare material for publication: *SHELXTL* and *PLATON* (Spek, 2009 ▶).

## Supplementary Material

Crystal structure: contains datablock(s) global, I. DOI: 10.1107/S1600536812032631/hb6903sup1.cif

Structure factors: contains datablock(s) I. DOI: 10.1107/S1600536812032631/hb6903Isup2.hkl

Supplementary material file. DOI: 10.1107/S1600536812032631/hb6903Isup3.cml

Additional supplementary materials:  crystallographic information; 3D view; checkCIF report

## Figures and Tables

**Table 1:** Hydrogen-bond geometry (Å, °)

*D*—H⋯*A*	*D*—H	H⋯*A*	*D*⋯*A*	*D*—H⋯*A*
N2*A*—H1N2⋯N1*B* ^i^	0.84	2.28	3.114 (2)	175
N2*B*—H2N2⋯N1*A* ^ii^	0.82	2.21	3.035 (2)	176
C3*A*—H3*AA*⋯O1*B* ^iii^	0.95	2.58	3.208 (2)	124
C4*A*—H4*AA*⋯O1*A*	0.95	2.22	2.832 (3)	121
C10*A*—H10*A*⋯O1*A* ^iv^	0.95	2.49	3.422 (3)	169
C4*B*—H4*BA*⋯O1*B*	0.95	2.25	2.846 (3)	120

Symmetry codes: (i) 
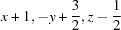
; (ii) 
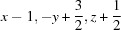
; (iii) 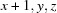; (iv) 
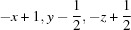
.

**Fig. 1.**
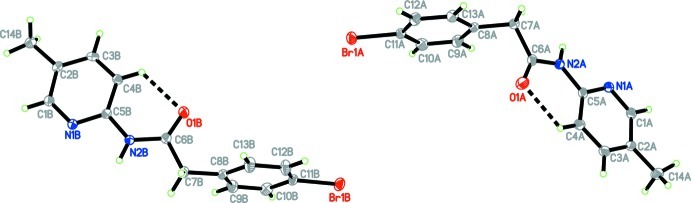
The molecular structure of the title compound, showing 50% probability displacement ellipsoids. Intramolecular hydrogen bonds are shown as dashed lines.

**Fig. 2.**
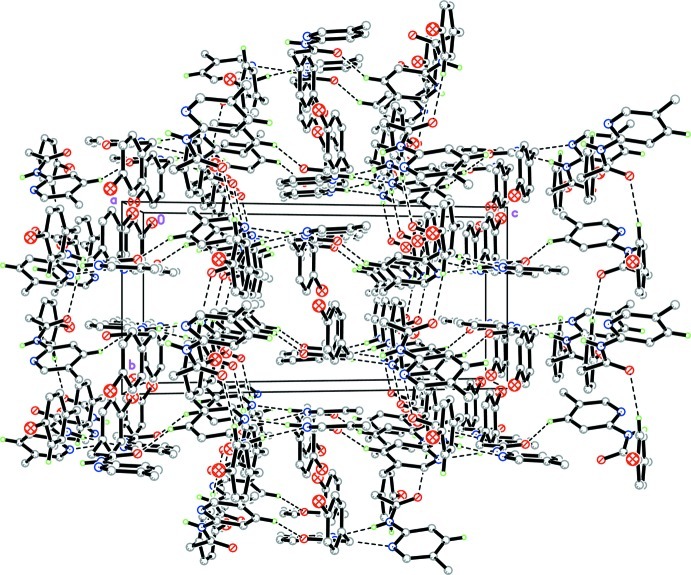
The crystal packing of the title compound, viewed along the a axis. H atoms not involved in the intermolecular interactions (dashed lines) have been omitted for clarity.

## supplementary crystallographic information

### Comment

In continuation of our work on synthesis of amides (Fun *et al.*, 2012*a*), we report herein the crystal structure of the title compound (I).

The asymmetric unit of the title compound (I) consists of two crystallographically independent molecules (A and B) as shown in Fig. 1. In both molecules, the pyridine (N1A/C1A–C5A and N1B/C1B–C5B) rings are essentially planar with maximum deviations of 0.013 (2) Å at N1A atom and 0.004 (2) Å at C2B and C5B atoms. The dihedral angle between the pyridine ring and bromo-substituted benzene ring (C8–C13) is 87.99 (9) in molecule A and 84.28 (9)° in molecule B. An intramolecular C—H···O interaction (Table 1), generates an S(6) ring motif (Bernstein *et al.*, 1995) in each molecule. The bond lengths and angles are comparable to those in a related structure (Fun *et al.*, 2012*b*).

In the crystal (Fig. 2), the molecules are linked *via* N2A—H1N2···N1B, N2B—H2N2···N1A, C3A—H3AA···O1B and C10A—H10A···O1A hydrogen bonds (Table 1) into a three-dimensional network. The crystal structure also features weak π—π interactions between bromo-substituted benzene rings (C8A–C13A and C8B–C13B) [centroid–centroid distance = 3.6829 (12) Å; *x*, 3/2 - *y*, -1/2 + *z*].

### Experimental

4-Bromophenylacetic acid (0.213 g, 1 mmol), 2-amino-5-methylpyridine (0.108 g, 1 mmol) and 1-ethyl-3-(3-dimethylaminopropyl)-carbodiimide hydrochloride (1.0 g, 0.01 mol) were dissolved in dichloromethane (20 ml). The mixture was stirred in presence of triethylamine at 273 K for about 3 h. The contents were poured into 100 ml of ice-cold aqueous hydrochloric acid with stirring which was extracted thrice with dichloromethane. Organic layer was washed with saturated NaHCO_3_ solution and brine solution, dried and concentrated under reduced pressure to give the title compound (I). Colourless blocks were grown from dichloromethane solution by the slow evaporation method (m.p. 459–461 K).

### Refinement

H1N2 and H2N2 atoms were located from the difference map and were fixed at their found positions with *U*_iso_(H) = 1.2 *U*_eq_(N). [N—H = 0.8222 and 0.8358 Å]. The remaining H atoms were positioned geometrically [C—H = 0.9500, 0.9800 and 0.9900 Å] with *U*_iso_(H) = 1.2 or 1.5*U*_eq_(C). A rotating group model was applied to the methyl groups.In the final refinement, two outliers (500) and (002) were omitted.

### Crystal data

**Table:**

C_14_H_13_BrN_2_O	*F*(000) = 1232
*M**_r_* = 305.17	*D*_x_ = 1.577 Mg m^−^^3^
Monoclinic, *P*2_1_/*c*	Mo *K*α radiation, λ = 0.71073 Å
Hall symbol: -P 2ybc	Cell parameters from 7856 reflections
*a* = 14.0086 (16) Å	θ = 2.4–29.9°
*b* = 9.4215 (11) Å	µ = 3.19 mm^−^^1^
*c* = 20.610 (2) Å	*T* = 100 K
β = 109.040 (2)°	Block, colourless
*V* = 2571.3 (5) Å^3^	0.35 × 0.31 × 0.16 mm
*Z* = 8	

### Data collection

**Table:**

Bruker APEX DUO CCD diffractometer	7539 independent reflections
Radiation source: fine-focus sealed tube	5774 reflections with *I* > 2σ(*I*)
Graphite monochromator	*R*_int_ = 0.049
φ and ω scans	θ_max_ = 30.1°, θ_min_ = 2.2°
Absorption correction: multi-scan (*SADABS*; Bruker, 2009)	*h* = −19→19
*T*_min_ = 0.404, *T*_max_ = 0.626	*k* = −13→11
28356 measured reflections	*l* = −29→29

### Refinement

**Table:**

Refinement on *F*^2^	Primary atom site location: structure-invariant direct methods
Least-squares matrix: full	Secondary atom site location: difference Fourier map
*R*[*F*^2^ > 2σ(*F*^2^)] = 0.033	Hydrogen site location: inferred from neighbouring sites
*wR*(*F*^2^) = 0.068	H-atom parameters constrained
*S* = 1.03	*w* = 1/[σ^2^(*F*_o_^2^) + (0.0229*P*)^2^ + 0.7284*P*] where *P* = (*F*_o_^2^ + 2*F*_c_^2^)/3
7539 reflections	(Δ/σ)_max_ = 0.001
327 parameters	Δρ_max_ = 0.72 e Å^−^^3^
0 restraints	Δρ_min_ = −0.84 e Å^−^^3^

### Special details

**Table:**

Experimental. The crystal was placed in the cold stream of an Oxford Cryosystems Cobra open-flow nitrogen cryostat (Cosier & Glazer, 1986) operating at 100 (1) K.
Geometry. All e.s.d.'s (except the e.s.d. in the dihedral angle between two l.s. planes) are estimated using the full covariance matrix. The cell e.s.d.'s are taken into account individually in the estimation of e.s.d.'s in distances, angles and torsion angles; correlations between e.s.d.'s in cell parameters are only used when they are defined by crystal symmetry. An approximate (isotropic) treatment of cell e.s.d.'s is used for estimating e.s.d.'s involving l.s. planes.
Refinement. Refinement of *F*^2^ against ALL reflections. The weighted *R*-factor *wR* and goodness of fit *S* are based on *F*^2^, conventional *R*-factors *R* are based on *F*, with *F* set to zero for negative *F*^2^. The threshold expression of *F*^2^ > σ(*F*^2^) is used only for calculating *R*-factors(gt) *etc*. and is not relevant to the choice of reflections for refinement. *R*-factors based on *F*^2^ are statistically about twice as large as those based on *F*, and *R*-factors based on ALL data will be even larger.

### Fractional atomic coordinates and isotropic or equivalent isotropic displacement parameters (Å^2^)

**Table:**

	*x*	*y*	*z*	*U*_iso_*/*U*_eq_	
Br1A	0.195964 (14)	0.81673 (2)	0.236716 (10)	0.02478 (6)	
O1A	0.68992 (10)	0.87227 (17)	0.26779 (7)	0.0239 (3)	
N1A	0.88325 (11)	0.63139 (18)	0.19256 (7)	0.0166 (3)	
N2A	0.73682 (11)	0.74607 (18)	0.18857 (7)	0.0155 (3)	
H1N2	0.7195	0.7183	0.1479	0.019*	
C1A	0.97524 (14)	0.5797 (2)	0.22730 (9)	0.0178 (4)	
H1AA	1.0100	0.5287	0.2021	0.021*	
C2A	1.02300 (14)	0.5955 (2)	0.29725 (9)	0.0170 (4)	
C3A	0.97107 (14)	0.6710 (2)	0.33286 (9)	0.0182 (4)	
H3AA	1.0015	0.6871	0.3807	0.022*	
C4A	0.87506 (14)	0.7233 (2)	0.29916 (9)	0.0176 (4)	
H4AA	0.8384	0.7735	0.3233	0.021*	
C5A	0.83407 (13)	0.6999 (2)	0.22894 (9)	0.0150 (4)	
C6A	0.67342 (13)	0.8314 (2)	0.20897 (9)	0.0161 (4)	
C7A	0.57883 (13)	0.8776 (2)	0.15194 (9)	0.0182 (4)	
H7AA	0.5863	0.9777	0.1399	0.022*	
H7AB	0.5710	0.8189	0.1107	0.022*	
C8A	0.48535 (13)	0.8631 (2)	0.17273 (9)	0.0154 (4)	
C9A	0.44796 (15)	0.7304 (2)	0.18061 (10)	0.0216 (4)	
H9AA	0.4820	0.6482	0.1729	0.026*	
C10A	0.36231 (15)	0.7145 (2)	0.19943 (10)	0.0221 (4)	
H10A	0.3379	0.6227	0.2049	0.026*	
C11A	0.31300 (14)	0.8351 (2)	0.21015 (9)	0.0178 (4)	
C12A	0.34796 (14)	0.9688 (2)	0.20254 (10)	0.0206 (4)	
H12A	0.3133	1.0507	0.2099	0.025*	
C13A	0.43454 (14)	0.9824 (2)	0.18395 (9)	0.0184 (4)	
H13A	0.4592	1.0743	0.1789	0.022*	
C14A	1.12626 (15)	0.5339 (2)	0.33246 (10)	0.0244 (4)	
H14A	1.1567	0.5051	0.2979	0.037*	
H14B	1.1202	0.4511	0.3596	0.037*	
H14C	1.1690	0.6055	0.3628	0.037*	
Br1B	0.362638 (14)	0.54031 (2)	0.494541 (10)	0.02378 (6)	
O1B	−0.09186 (10)	0.80166 (16)	0.45686 (6)	0.0230 (3)	
N1B	−0.33380 (11)	0.83860 (17)	0.53410 (7)	0.0154 (3)	
N2B	−0.16779 (11)	0.82936 (17)	0.53881 (7)	0.0155 (3)	
H2N2	−0.1559	0.8362	0.5805	0.019*	
C1B	−0.43308 (14)	0.8465 (2)	0.49897 (9)	0.0170 (4)	
H1BA	−0.4785	0.8476	0.5246	0.020*	
C2B	−0.47401 (13)	0.8532 (2)	0.42804 (9)	0.0162 (4)	
C3B	−0.40554 (14)	0.8531 (2)	0.39206 (9)	0.0167 (4)	
H3BA	−0.4296	0.8584	0.3434	0.020*	
C4B	−0.30301 (14)	0.8453 (2)	0.42630 (9)	0.0154 (4)	
H4BA	−0.2561	0.8453	0.4018	0.019*	
C5B	−0.27001 (13)	0.8375 (2)	0.49800 (9)	0.0141 (4)	
C6B	−0.08622 (14)	0.8118 (2)	0.51717 (9)	0.0162 (4)	
C7B	0.01452 (13)	0.8088 (2)	0.57477 (9)	0.0200 (4)	
H7BA	0.0340	0.9071	0.5905	0.024*	
H7BB	0.0067	0.7548	0.6139	0.024*	
C8B	0.09776 (13)	0.7428 (2)	0.55364 (9)	0.0164 (4)	
C9B	0.11021 (14)	0.5975 (2)	0.55405 (10)	0.0213 (4)	
H9BA	0.0643	0.5381	0.5667	0.026*	
C10B	0.18826 (15)	0.5365 (2)	0.53639 (10)	0.0215 (4)	
H10B	0.1962	0.4363	0.5370	0.026*	
C11B	0.25450 (14)	0.6235 (2)	0.51780 (9)	0.0176 (4)	
C12B	0.24397 (15)	0.7683 (2)	0.51692 (11)	0.0250 (5)	
H12B	0.2899	0.8274	0.5042	0.030*	
C13B	0.16501 (15)	0.8274 (2)	0.53492 (11)	0.0240 (4)	
H13B	0.1572	0.9276	0.5343	0.029*	
C14B	−0.58628 (14)	0.8603 (2)	0.39206 (10)	0.0218 (4)	
H14D	−0.6208	0.8732	0.4261	0.033*	
H14E	−0.6092	0.7719	0.3667	0.033*	
H14F	−0.6019	0.9405	0.3600	0.033*	

### Atomic displacement parameters (Å^2^)

**Table:**

	*U*^11^	*U*^22^	*U*^33^	*U*^12^	*U*^13^	*U*^23^
Br1A	0.01468 (9)	0.04023 (14)	0.01992 (9)	−0.00378 (9)	0.00632 (7)	−0.00040 (9)
O1A	0.0213 (7)	0.0316 (9)	0.0176 (6)	0.0071 (6)	0.0048 (5)	−0.0048 (6)
N1A	0.0160 (7)	0.0188 (9)	0.0158 (7)	0.0023 (7)	0.0063 (6)	0.0012 (6)
N2A	0.0140 (7)	0.0203 (9)	0.0122 (7)	0.0003 (7)	0.0041 (6)	−0.0015 (6)
C1A	0.0177 (9)	0.0193 (11)	0.0185 (8)	0.0039 (8)	0.0088 (7)	0.0017 (7)
C2A	0.0158 (9)	0.0173 (11)	0.0176 (8)	0.0003 (8)	0.0051 (7)	0.0038 (7)
C3A	0.0188 (9)	0.0204 (11)	0.0148 (8)	−0.0016 (8)	0.0045 (7)	0.0002 (7)
C4A	0.0186 (9)	0.0193 (11)	0.0157 (8)	0.0018 (8)	0.0066 (7)	−0.0007 (7)
C5A	0.0148 (8)	0.0153 (10)	0.0156 (8)	−0.0013 (7)	0.0058 (7)	0.0002 (7)
C6A	0.0148 (8)	0.0159 (10)	0.0187 (8)	−0.0004 (7)	0.0068 (7)	0.0010 (7)
C7A	0.0167 (9)	0.0212 (11)	0.0169 (8)	0.0042 (8)	0.0058 (7)	0.0034 (7)
C8A	0.0124 (8)	0.0188 (11)	0.0142 (8)	0.0019 (7)	0.0031 (7)	0.0010 (7)
C9A	0.0216 (10)	0.0177 (11)	0.0265 (10)	0.0044 (8)	0.0092 (8)	0.0011 (8)
C10A	0.0231 (10)	0.0175 (11)	0.0254 (10)	−0.0022 (8)	0.0076 (8)	0.0026 (8)
C11A	0.0137 (8)	0.0243 (12)	0.0147 (8)	−0.0021 (8)	0.0034 (7)	0.0000 (8)
C12A	0.0198 (9)	0.0196 (11)	0.0233 (9)	0.0042 (8)	0.0082 (8)	−0.0025 (8)
C13A	0.0186 (9)	0.0140 (11)	0.0218 (9)	−0.0012 (8)	0.0056 (7)	−0.0003 (7)
C14A	0.0207 (10)	0.0280 (13)	0.0227 (9)	0.0080 (9)	0.0048 (8)	0.0043 (9)
Br1B	0.01786 (10)	0.03056 (13)	0.02292 (10)	0.00614 (9)	0.00664 (7)	−0.00641 (8)
O1B	0.0181 (7)	0.0362 (10)	0.0154 (6)	0.0040 (6)	0.0063 (5)	−0.0024 (6)
N1B	0.0140 (7)	0.0177 (9)	0.0145 (7)	0.0014 (6)	0.0047 (6)	0.0001 (6)
N2B	0.0137 (7)	0.0205 (9)	0.0119 (6)	0.0017 (6)	0.0037 (6)	0.0000 (6)
C1B	0.0158 (9)	0.0181 (11)	0.0179 (8)	0.0014 (7)	0.0068 (7)	0.0016 (7)
C2B	0.0149 (9)	0.0141 (10)	0.0177 (8)	0.0015 (7)	0.0027 (7)	0.0006 (7)
C3B	0.0205 (9)	0.0148 (10)	0.0127 (8)	−0.0002 (8)	0.0025 (7)	0.0014 (7)
C4B	0.0181 (9)	0.0142 (10)	0.0144 (8)	0.0005 (7)	0.0058 (7)	0.0004 (7)
C5B	0.0131 (8)	0.0125 (10)	0.0160 (8)	0.0012 (7)	0.0036 (7)	0.0003 (7)
C6B	0.0152 (9)	0.0164 (10)	0.0171 (8)	0.0015 (7)	0.0054 (7)	0.0005 (7)
C7B	0.0140 (9)	0.0288 (12)	0.0167 (8)	0.0013 (8)	0.0045 (7)	−0.0028 (8)
C8B	0.0124 (8)	0.0219 (11)	0.0138 (8)	0.0009 (8)	0.0028 (7)	−0.0002 (7)
C9B	0.0194 (9)	0.0214 (12)	0.0241 (9)	−0.0053 (8)	0.0084 (8)	0.0005 (8)
C10B	0.0230 (10)	0.0146 (11)	0.0260 (10)	0.0002 (8)	0.0065 (8)	−0.0007 (8)
C11B	0.0148 (9)	0.0210 (11)	0.0165 (8)	0.0044 (8)	0.0043 (7)	−0.0026 (7)
C12B	0.0224 (10)	0.0219 (12)	0.0366 (11)	−0.0009 (9)	0.0176 (9)	0.0026 (9)
C13B	0.0241 (10)	0.0153 (11)	0.0368 (11)	0.0021 (8)	0.0156 (9)	0.0016 (9)
C14B	0.0165 (9)	0.0280 (12)	0.0182 (9)	0.0020 (8)	0.0021 (7)	0.0004 (8)

### Geometric parameters (Å, º)

**Table:**

Br1A—C11A	1.8982 (18)	Br1B—C11B	1.9007 (18)
O1A—C6A	1.220 (2)	O1B—C6B	1.223 (2)
N1A—C5A	1.338 (2)	N1B—C5B	1.336 (2)
N1A—C1A	1.345 (2)	N1B—C1B	1.344 (2)
N2A—C6A	1.362 (2)	N2B—C6B	1.365 (2)
N2A—C5A	1.413 (2)	N2B—C5B	1.406 (2)
N2A—H1N2	0.8358	N2B—H2N2	0.8222
C1A—C2A	1.385 (2)	C1B—C2B	1.387 (2)
C1A—H1AA	0.9500	C1B—H1BA	0.9500
C2A—C3A	1.386 (3)	C2B—C3B	1.391 (2)
C2A—C14A	1.507 (3)	C2B—C14B	1.506 (3)
C3A—C4A	1.387 (3)	C3B—C4B	1.380 (3)
C3A—H3AA	0.9500	C3B—H3BA	0.9500
C4A—C5A	1.389 (2)	C4B—C5B	1.399 (2)
C4A—H4AA	0.9500	C4B—H4BA	0.9500
C6A—C7A	1.520 (3)	C6B—C7B	1.519 (3)
C7A—C8A	1.511 (2)	C7B—C8B	1.506 (2)
C7A—H7AA	0.9900	C7B—H7BA	0.9900
C7A—H7AB	0.9900	C7B—H7BB	0.9900
C8A—C9A	1.385 (3)	C8B—C9B	1.380 (3)
C8A—C13A	1.390 (3)	C8B—C13B	1.382 (3)
C9A—C10A	1.384 (3)	C9B—C10B	1.385 (3)
C9A—H9AA	0.9500	C9B—H9BA	0.9500
C10A—C11A	1.384 (3)	C10B—C11B	1.383 (3)
C10A—H10A	0.9500	C10B—H10B	0.9500
C11A—C12A	1.379 (3)	C11B—C12B	1.372 (3)
C12A—C13A	1.392 (3)	C12B—C13B	1.392 (3)
C12A—H12A	0.9500	C12B—H12B	0.9500
C13A—H13A	0.9500	C13B—H13B	0.9500
C14A—H14A	0.9800	C14B—H14D	0.9800
C14A—H14B	0.9800	C14B—H14E	0.9800
C14A—H14C	0.9800	C14B—H14F	0.9800
			
C5A—N1A—C1A	117.09 (15)	C5B—N1B—C1B	117.56 (15)
C6A—N2A—C5A	126.89 (15)	C6B—N2B—C5B	127.49 (15)
C6A—N2A—H1N2	119.3	C6B—N2B—H2N2	116.4
C5A—N2A—H1N2	113.7	C5B—N2B—H2N2	116.1
N1A—C1A—C2A	124.66 (17)	N1B—C1B—C2B	124.73 (16)
N1A—C1A—H1AA	117.7	N1B—C1B—H1BA	117.6
C2A—C1A—H1AA	117.7	C2B—C1B—H1BA	117.6
C1A—C2A—C3A	116.59 (17)	C1B—C2B—C3B	116.25 (16)
C1A—C2A—C14A	121.43 (17)	C1B—C2B—C14B	121.83 (16)
C3A—C2A—C14A	121.98 (17)	C3B—C2B—C14B	121.92 (16)
C2A—C3A—C4A	120.49 (17)	C4B—C3B—C2B	120.74 (16)
C2A—C3A—H3AA	119.8	C4B—C3B—H3BA	119.6
C4A—C3A—H3AA	119.8	C2B—C3B—H3BA	119.6
C3A—C4A—C5A	117.97 (17)	C3B—C4B—C5B	118.22 (16)
C3A—C4A—H4AA	121.0	C3B—C4B—H4BA	120.9
C5A—C4A—H4AA	121.0	C5B—C4B—H4BA	120.9
N1A—C5A—C4A	123.14 (17)	N1B—C5B—C4B	122.49 (16)
N1A—C5A—N2A	113.09 (15)	N1B—C5B—N2B	113.76 (15)
C4A—C5A—N2A	123.77 (16)	C4B—C5B—N2B	123.75 (16)
O1A—C6A—N2A	124.16 (17)	O1B—C6B—N2B	123.91 (17)
O1A—C6A—C7A	120.87 (17)	O1B—C6B—C7B	121.87 (16)
N2A—C6A—C7A	114.96 (15)	N2B—C6B—C7B	114.21 (15)
C8A—C7A—C6A	111.88 (14)	C8B—C7B—C6B	113.03 (15)
C8A—C7A—H7AA	109.2	C8B—C7B—H7BA	109.0
C6A—C7A—H7AA	109.2	C6B—C7B—H7BA	109.0
C8A—C7A—H7AB	109.2	C8B—C7B—H7BB	109.0
C6A—C7A—H7AB	109.2	C6B—C7B—H7BB	109.0
H7AA—C7A—H7AB	107.9	H7BA—C7B—H7BB	107.8
C9A—C8A—C13A	118.47 (17)	C9B—C8B—C13B	118.53 (18)
C9A—C8A—C7A	120.71 (17)	C9B—C8B—C7B	121.10 (17)
C13A—C8A—C7A	120.82 (18)	C13B—C8B—C7B	120.35 (19)
C10A—C9A—C8A	121.74 (19)	C8B—C9B—C10B	121.28 (18)
C10A—C9A—H9AA	119.1	C8B—C9B—H9BA	119.4
C8A—C9A—H9AA	119.1	C10B—C9B—H9BA	119.4
C9A—C10A—C11A	118.64 (19)	C11B—C10B—C9B	119.05 (19)
C9A—C10A—H10A	120.7	C11B—C10B—H10B	120.5
C11A—C10A—H10A	120.7	C9B—C10B—H10B	120.5
C12A—C11A—C10A	121.15 (17)	C12B—C11B—C10B	120.95 (18)
C12A—C11A—Br1A	119.22 (14)	C12B—C11B—Br1B	119.86 (15)
C10A—C11A—Br1A	119.63 (15)	C10B—C11B—Br1B	119.18 (15)
C11A—C12A—C13A	119.29 (18)	C11B—C12B—C13B	119.04 (19)
C11A—C12A—H12A	120.4	C11B—C12B—H12B	120.5
C13A—C12A—H12A	120.4	C13B—C12B—H12B	120.5
C8A—C13A—C12A	120.71 (19)	C8B—C13B—C12B	121.1 (2)
C8A—C13A—H13A	119.6	C8B—C13B—H13B	119.4
C12A—C13A—H13A	119.6	C12B—C13B—H13B	119.4
C2A—C14A—H14A	109.5	C2B—C14B—H14D	109.5
C2A—C14A—H14B	109.5	C2B—C14B—H14E	109.5
H14A—C14A—H14B	109.5	H14D—C14B—H14E	109.5
C2A—C14A—H14C	109.5	C2B—C14B—H14F	109.5
H14A—C14A—H14C	109.5	H14D—C14B—H14F	109.5
H14B—C14A—H14C	109.5	H14E—C14B—H14F	109.5
			
C5A—N1A—C1A—C2A	−1.6 (3)	C5B—N1B—C1B—C2B	0.2 (3)
N1A—C1A—C2A—C3A	−0.3 (3)	N1B—C1B—C2B—C3B	−0.7 (3)
N1A—C1A—C2A—C14A	179.84 (19)	N1B—C1B—C2B—C14B	179.30 (19)
C1A—C2A—C3A—C4A	1.8 (3)	C1B—C2B—C3B—C4B	0.6 (3)
C14A—C2A—C3A—C4A	−178.39 (19)	C14B—C2B—C3B—C4B	−179.46 (19)
C2A—C3A—C4A—C5A	−1.2 (3)	C2B—C3B—C4B—C5B	0.1 (3)
C1A—N1A—C5A—C4A	2.3 (3)	C1B—N1B—C5B—C4B	0.5 (3)
C1A—N1A—C5A—N2A	−177.92 (16)	C1B—N1B—C5B—N2B	179.86 (17)
C3A—C4A—C5A—N1A	−0.9 (3)	C3B—C4B—C5B—N1B	−0.6 (3)
C3A—C4A—C5A—N2A	179.32 (18)	C3B—C4B—C5B—N2B	−179.92 (18)
C6A—N2A—C5A—N1A	−172.21 (18)	C6B—N2B—C5B—N1B	172.75 (18)
C6A—N2A—C5A—C4A	7.6 (3)	C6B—N2B—C5B—C4B	−7.9 (3)
C5A—N2A—C6A—O1A	−5.5 (3)	C5B—N2B—C6B—O1B	0.6 (3)
C5A—N2A—C6A—C7A	172.96 (17)	C5B—N2B—C6B—C7B	179.44 (18)
O1A—C6A—C7A—C8A	−47.0 (3)	O1B—C6B—C7B—C8B	−20.2 (3)
N2A—C6A—C7A—C8A	134.48 (18)	N2B—C6B—C7B—C8B	160.96 (17)
C6A—C7A—C8A—C9A	−70.1 (2)	C6B—C7B—C8B—C9B	−83.0 (2)
C6A—C7A—C8A—C13A	110.3 (2)	C6B—C7B—C8B—C13B	98.5 (2)
C13A—C8A—C9A—C10A	−0.2 (3)	C13B—C8B—C9B—C10B	0.2 (3)
C7A—C8A—C9A—C10A	−179.82 (18)	C7B—C8B—C9B—C10B	−178.28 (17)
C8A—C9A—C10A—C11A	0.4 (3)	C8B—C9B—C10B—C11B	−0.2 (3)
C9A—C10A—C11A—C12A	−0.1 (3)	C9B—C10B—C11B—C12B	0.2 (3)
C9A—C10A—C11A—Br1A	−179.49 (14)	C9B—C10B—C11B—Br1B	179.29 (14)
C10A—C11A—C12A—C13A	−0.2 (3)	C10B—C11B—C12B—C13B	−0.2 (3)
Br1A—C11A—C12A—C13A	179.15 (14)	Br1B—C11B—C12B—C13B	−179.23 (16)
C9A—C8A—C13A—C12A	−0.1 (3)	C9B—C8B—C13B—C12B	−0.1 (3)
C7A—C8A—C13A—C12A	179.47 (17)	C7B—C8B—C13B—C12B	178.35 (18)
C11A—C12A—C13A—C8A	0.3 (3)	C11B—C12B—C13B—C8B	0.1 (3)

### Hydrogen-bond geometry (Å, º)

**Table:**

*D*—H···*A*	*D*—H	H···*A*	*D*···*A*	*D*—H···*A*
N2*A*—H1*N*2···N1*B*^i^	0.84	2.28	3.114 (2)	175
N2*B*—H2*N*2···N1*A*^ii^	0.82	2.21	3.035 (2)	176
C3*A*—H3*AA*···O1*B*^iii^	0.95	2.58	3.208 (2)	124
C4*A*—H4*AA*···O1*A*	0.95	2.22	2.832 (3)	121
C10*A*—H10*A*···O1*A*^iv^	0.95	2.49	3.422 (3)	169
C4*B*—H4*BA*···O1*B*	0.95	2.25	2.846 (3)	120

Symmetry codes: (i) *x*+1, −*y*+3/2, *z*−1/2; (ii) *x*−1, −*y*+3/2, *z*+1/2; (iii) *x*+1, *y*, *z*; (iv) −*x*+1, *y*−1/2, −*z*+1/2.
